# Broadening the coating applications of sustainable materials by reinforcing epoxidized corn oil with single-walled carbon nanotubes

**DOI:** 10.1007/s11356-024-33702-2

**Published:** 2024-05-22

**Authors:** Mădălina Ioana Necolau, Iulia Nicoleta Radu, Brînduşa Bălănucă, Adriana Nicoleta Frone, Celina Maria Damian

**Affiliations:** 1 Advanced Polymer Materials Group, National University of Science and Technology, Politehnica Bucharest 1-7 Gh. Polizu Street, 011061 Bucharest, Romania; 2 Department of Organic Chemistry “C. Nenitescu, National University of Science and Technology, Politehnica Bucharest 1-7 Gh. Polizu Street, 011061 Bucharest, Romania; 3 National Institute for Research & Development in Chemistry and Petrochemistry-ICECHIM, 202 Spl. Independentei, 060021 Bucharest, Romania

**Keywords:** Bio-based epoxy, Functionalized carbon nanotubes, Sustainable nanocomposites, Thermal properties, Bio-epoxy coatings, Swelling

## Abstract

In the global context of environmental awareness, the present research proposes a sustainable alternative to the widely used petroleum-based epoxy coatings. Epoxidized corn oil (ECO) was tested as potential matrix for advanced nanocomposite coating materials reinforced with 0.25 to 1 wt.% single-walled carbon nanotubes (SW) with carboxyl and amide functionalities. The elemental composition of the epoxy networks was monitored by XPS, showing the increase of O/C ratio to 0.387 when carboxyl-functionalized SW are added. To achieve sustainable composite materials, citric acid was used as curing agent, as a substitute for conventional counterparts. The influence of both surface functional groups and concentration of SW was evaluated through structural and thermo-mechanical analysis. The progressive increase of the DSC enthalpy for SW formulated systems indicates a possible pattern for specific interactions within the bio-based epoxy translated by adjusted activation energy. For 1% neat SW addition, the Ea values decreased to 46 kJ/mol in comparison with 53 kJ/mol calculated for neat epoxy. Furthermore, the -COOH groups from SW nanostructures exerted a strong influence over the mechanical performance of bio-epoxy networks, improving the crosslinking density with ~ 60% and twofold the storage modulus value. Accordingly, by gradual addition of SW-COOH filler within the ECO-based formulations, a very consistent behaviour in seawater was noted, with a 28% decreased value for the absorption degree.

**Graphical Abstract:**

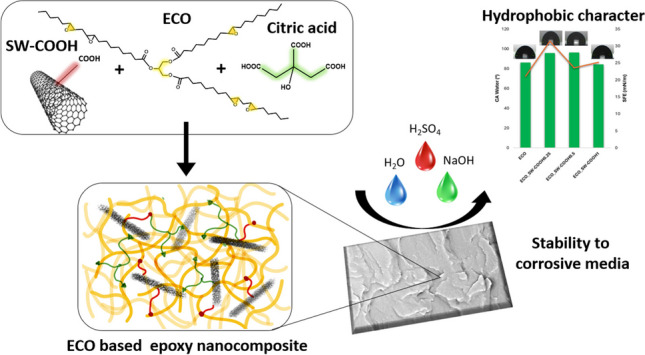

## Introduction

Corrosion represents one of the most concerning processes driven by environmental factors which affect metallic substrates and consequently end-use industries such as automotive or marine (Abdi et al. 2022; Chami et al. 2021). Epoxy resins are currently the most preferred materials for a variety of anticorrosion coatings, which can easily be applied on a large range of substrates (Abdus Samad et al. 2023). The epoxy-based coating industry is witnessing a significant growth driven by the increasing demand of sustainable and advanced materials with increased pot-life. Based on their unique capacity to generate tough, and chemically resistant materials, epoxy coating is spread in a wide range of industries such as aircraft, automotive, electronic, or naval (Aparna et al. 2022) where durable and efficient materials are required. Furthermore, a sustainable approach within this area must be driven by the aim to obtain analogous materials with suitable properties.

When it comes to coatings industry, whether they are deposited on a metallic or non-metallic support, petroleum-based epoxy resins are amongst the most used materials due to their demonstrated excellent performances (Verma et al. 2020; Wei et al. 2020). Not only the facile processability, strong adhesion, strength (thermal or electrical), and stiffness make them an ideal anticorrosion polymeric matrix but also the exceptional resistance to the organic solvents and other different corrosive media (Dagdag et al. 2022; Miyagawa et al. 2005).

Despite all these, the high costs of these petroleum-based materials, the associated toxicity during production, and also to their end-of-life disposal which generate high undegradable waste quantities are the most concerning problems that drive the industry to search for more ecofriendly alternatives and sustainable technologies. In recent years, the development of new polymers from renewable resources has gained increasing attention, which has led to the emergence of rational and sustainable synthetic routes, through biomass processing, generating bio-based products that can achieve the properties of conventional ones (da Silva et al. 2022; Gonçalves et al. 2022; Kumar et al. 2018a, b; Kumar et al. 2018a, b). Bio-based epoxy resins derived from vegetable oils (Agbo et al. 2023; Díez-Pascual and Rahdar 2021; Marriam et al. 2023; Mustapha et al. 2019), lignin and lignin derivatives (Bass and Epps 2021; Lu and Gu 2023; Sreejaya et al. 2022) phenolic and polyphenolic structures, or saccharides (Ebrahimnezhad-Khaljiri and Ghadi 2024; Wan et al. 2020; Zhang et al. 2022) were developed and studied, thus widening the platform of materials adaptable to different industries, especially for anticorrosion protection coatings. However, as the availability of bio-based thermoplastic is quite large, the bio-based thermosetting field is still limited by the low physical and mechanical properties of the polymers derived from renewable resources; thus, this field represents a real challenge for both researchers and industry. Therefore, one of the many attempts which have been made to improve the thermal, mechanical, and anticorrosion performances of the bio-based polymeric materials consists in the development of novel (nano)composites with improved properties (Dagdag et al. 2023; Miyagawa et al. 2005). For this, different epoxides from renewable resources were formulated with synthetic or natural reinforcing agents, neat ones or their functionalized derivatives (graphene, carbon nanotubes, lignin, nanocellulose, clays, and so on) (Balanuca et al. 2023; Dyachkova et al. 2023; Li et al. 2022; Liu et al. 2021; Randis et al. 2023; Wu et al. 2021). Regarding anticorrosion protection, high moisture resistance translated by a hydrophobic behaviour is another envisaged feature for the developed protective layers (Aparna et al. 2022; Ashok Kumar et al. 2022).

Carbon nanotubes possess excellent chemical stability, superior thermo-mechanical features and high surface area due to their distinctive one-dimensional chemical structure. Composites based on CNTs have good barrier properties under critical environmental conditions and can produce protective layers over metallic substrates (Calabrese et al. 2020; Rui et al. 2019). Moreover, the presence of CNT within epoxy nanocomposites improve the adhesion properties and decreased the porosity of the polymeric matrix minimizing thus the possibility for the formation of micro voids or other structural defects which decrease the barrier properties (Ganguli et al. 2006, 2005).

A recent study developed an intertwined hybrid nano-reinforcing agent based on CNT and copper and used to prepare epoxy superhydrophobic and anticorrosive coatings (Zhu et al. 2023). When the corrosion current density was analysed, it reached 9.55 × 10^−8^ A/cm^2^ denoting outstanding corrosion resistance. Hiremath et al. has recently evaluated the wear resistance of CNT reinforced bio-epoxy system based on cashew nut oil and showed improved wear resistance by increasing filler content from 0.25 to 0.75% (Hiremath et al. 2023). A study conducted by Deyab demonstrated that the functionalization of CNT lead to significant improvements of the anticorrosion performances of epoxy-based nanocomposite coatings by increasing the compatibility between the polymeric matrix and the filler (Deyab and Awadallah 2020). The presence of additional functional groups introduced through acid treatment on the surface of the CNT led to superior anticorrosion performances of the final epoxy nanocomposite coating and also to better mechanical performances. CNT have recently been used to develop a pH-triggered self-inhibition nanofiller that can further be used in intelligent epoxy nanocomposites for anticorrosive applications (Cai et al. 2023). The CNT wrapped with tannic acid and cerium ions showed an increased dispersion within the polymeric matrix and led to a compact epoxy-based coating with excellent anticorrosive properties.

Epoxidized corn oil (ECO) represents a strong alternative as epoxy matrix for coating applications due to several associated advantages. In comparison with the high price for petroleum-based epoxy monomers, it is synthesized from an available raw material that can easily be extracted from globally extensive corn crops with a low price. The high chemical versatility which comes from the convenient unsaturation degree represents one of the main reasons for the conversion of corn oil into epoxy derivatives with a high epoxy functionalization degree. Based on this feature, great network properties can be achieved. However, this valuable resource was studied in very few research papers. Apart from that, generally the vegetable oil-based epoxy derivatives have shown great compatibility with different organic/inorganic/hybrid agents, as successful strategy to generate sustainable advanced materials. Up to now, there are a limited number of research papers focusing on the coating applications for epoxidized corn oil-based materials. A recent study successfully demonstrated the use of such thermoset for fire resistant applications; however, the anhydride used as crosslinking agent has corrosive and health hazardous effects (Cabo et al. 2021). Another study conducted by L. Rosu et al. studied the curing mechanism of epoxidized oil and DGEBA mixture with rosin derivatives and use of resulted materials as wood coatings, but only 20% wt. ECO was used (Rosu et al. 2021). Considering the sustainability objective of our research, the proposed systems cured with citric acid represent a strong alternative towards green materials, with convenient properties for the coating industry.

Based on the superior properties given by CNT and the promising perspectives within the conventional epoxy nanocomposites to develop advanced anticorrosive systems, extrapolation of this type of reinforcing mechanism was projected on the current study. In the discussed context, the current research deals with the property improvement of the sustainable and hydrophobic polymeric network of the epoxidized corn oil (ECO) through the incorporation of some optimized high-performance nano-reinforcing agents as amide- and carboxyl-functionalized single-walled carbon nanotubes (SW) that are well known for their superior thermal and mechanical features. Thus, the main objective of the present study was the development of different ECO-SW polymer nanomaterials as promising sustainable materials, through a thermal curing strategy using an efficient ecofriendly crosslinking agent, citric acid (CA) (Necolau et al. 2023; Sahoo et al. 2018). The epoxy curing reaction in the presence of the employed carbon nano-species was monitored, and the obtained materials were investigated regarding their structural, thermal, and thermo-mechanical characteristics. Also, the affinity for the aqueous environments (water and seawater) was observed for the ECO-based polymeric nanomaterials.

## Materials

Epoxidized corn oil (ECO) was obtained by our team through the epoxidation of a commercial corn oil, using literature reported experimental protocol involving in situ generation of peracetic acid (Balanuca et al. 2017; Necolau et al. 2023, 2022). The epoxy equivalent for ECO was calculated by using Eq. (1) and found to be 215 eq/mol, determined through HCl reaction of epoxy groups, followed by KOH titration (Li et al. 2007).

$$EE=\frac{m\cdot1000}{\left(V_0-V_1\right)\cdot0,1\cdot F},\left[g/echiv\right]$$

where *m* is the sample mass (g), *V*_0_ the volume of KOH for reference titration, *V*_1_ the volume of KOH for ECO sample titration, and *F* is the factor of 0.1 N KOH solution.

Citric acid (CA, 99 + %, from Alfa Aesar) and tetrahydrofuran (THF, ≥ 99.9% from Sigma-Aldrich) were used without any other treatments.

Single-walled carbon nanotube (SW), single-walled amide functionalized carbon nanotube (SW-am), and single-walled carboxylic acid functionalized carbon nanotube (SW-COOH) from Sigma-Aldrich were used as received as reinforcing agents.

## Methods

The ECO-based nanocomposites were synthesized by employing 0.25, 0.5, and 1 wt % of each reinforcing agent as follows: the nano-reinforcing agents (neat SW, SW-am, SW-COOH) were well dispersed in the ECO continuous phase by using a tip ultrasonicator (frequency of 100 kHz and amplitude of 30%) for 15 min.

ECO-based nanocomposites were crosslinked by considering a 1:1 stoichiometric ratio between the COOH groups (CA) and epoxy rings (ECO). CA was first solubilized in THF at 80 °C and then added to the ECO-SW blends and mixed for 5 min. The resulting mixtures were placed in Teflon moulds and cured for 3 h at 80 °C, 1 h at 100 °C, and 1 h at 120 °C. The synthesized samples were named along the manuscript as described in Table 1.

**Table 1:** Sample composition description and abbreviations

Matrix	Reinforcing agent	wt. %	Sample abbreviation
ECO-AC	-	-	ECO
ECO-AC	SW	0.25%	ECO_SW0.25%
ECO-AC	SW	0.5%	ECO_SW0.5%
ECO-AC	SW	1%	ECO_SW1%
ECO-AC	SW-am	0.25%	ECO_SW-am0.25%
ECO-AC	SW-am	0.5%	ECO_SW-am0.5%
ECO-AC	SW-am	1%	ECO_SW-am1%
ECO-AC	SW-COOH	0.25%	ECO_SW-COOH0.25%
ECO-AC	SW-COOH	0.5%	ECO_SW-COOH0.5%
ECO-AC	SW-COOH	1%	ECO_SW-COOH1%

## Characterization

*X-ray photoelectron spectrometry* (XPS) analysis was conducted on a K-Alpha spectrometer with a monochromatic Al Kα source (1486.6 eV) working in a vacuum base pressure of 2 × 10^−9^ mbar. Charging effects were compensated using a flood gun, and binding energy was calibrated by placing the C1s peak at 284.8 eV as an internal standard. Deconvolution of C 1 s peaks was performed by using a smart background algorithm with a convolved Gaussian − Lorentzian ratio. The pass energy for the survey spectra was set at 200 eV, while for the high-resolution C 1 s spectra registration it was 20 eV.

For *differential scanning calorimetry* (DSC), a Netzsch DSC 204 F1 Phoenix equipment was employed using heating rates of 5, 10, 15, and 20 °C/ min and inert nitrogen atmosphere (20 mL/min flow rate) heating from 20 to 300 °C. From the DSC data, the apparent activation energy (Ea) of the epoxy curing reaction was computed using Kissinger (1) and Ozawa (2) equations (Shih et al. 2022):

1 $$\mathbf{ln}\left(\frac{{\varvec{\beta}}}{{{{\varvec{T}}}_{{\varvec{P}}}}^{2}}\right)={\varvec{l}}{\varvec{n}}\frac{{\varvec{A}}{\varvec{R}}}{{\varvec{E}}{\varvec{a}}}-\frac{{\varvec{E}}{\varvec{a}}}{{\varvec{R}}{{\varvec{T}}}_{{\varvec{p}}}}$$

2 $${\varvec{l}}{\varvec{n}}\boldsymbol{ }{\varvec{\beta}}=-1.052{{\varvec{E}}}_{{\varvec{a}}}{\varvec{R}}{{\varvec{T}}}_{{\varvec{p}}}+{\varvec{C}}$$

where *β* is the heating rate (℃/min); Tp is the maximum temperature of the polymerization peak (K); *A* is the pre-exponential factor; *R* is the gas constant (*R* = 8.314 J/mol*K); Ea is the activation energy (KJ/mol); and *C* is a constant.

*Thermogravimetric analysis* (TGA) was performed using a Netzsch TG 209 F1 Libra equipment, from RT to 800 °C under nitrogen and synthetic air atmosphere with a heating rate of 10 °C/ min.

*Dynamic-mechanical analysis* (DMA) was done on a TRITEC 2000 B equipment in single cantilever bending mode at 1 Hz frequency in the temperature range of − 90 to 100 °C with a heating rate of 4 °C/min. Crosslinking density (υ_e_) was calculated for each studied system based on the DMA results, with the following equation (Necolau et al. 2022):

3 $${v}_{e}={E}{\prime}/3RT$$

where E′ is the storage modulus in the rubbery region at *T* = Tg + 30 and *T* and *R* correspond to the absolute temperature (K) and the ideal gas constant, respectively.

*Contact angle (CA) measurements* were performed with the aid of a Drop Shape Analyzer-DSA100 from Krüss Scientific GmbH using water and ethylene glycol as probing liquids at room temperature by using sessile drop method. Measurements were performed by placing a 2 μL solvent droplet on the sample surface, and the contact angle values were determined using the Young − Laplace equation in Advance software. The surface free energy for each system was calculated in the same software using the Young − Dupré and Fowkes equations which considered the polar work of adhesion and corresponding surface tensions of the liquids used for analysis.

The *absorption degree of water or seawater* (AD_water_/AD_NaCl_, %) was measured for the synthesized ECO-based polymeric composites, based on a standard method for water absorption (ASTMD570). For this, each sample was precisely weighted (m0) and then placed in the incubation medium (water or 3.5% concentration NaCl solution) where they were maintained for 7 days at room temperature. After 7 days, each specimen was extracted, the solvent excess was removed (filter paper), and the final weights were recorded (m1). The ADwater/ADNaCl values were calculated using the equation below:

4 $$AD\left(\%\right)=\frac{m1-m0}{m0}\times 100$$

The morphology of bio-epoxy-based nanocomposites was also analysed by *scanning electron microscopy (SEM)*. The samples were fractured in liquid nitrogen and then sputter-coated with gold and investigated by means of a Quanta 200 Environmental scanning electron microscope (FEI-Philips, USA) with a tungsten electron source, following a low vacuum mode, at an accelerating voltage of 30 kV.

## Results and discussions

The structural composition of the synthesized materials was assessed through elemental composition revealed by the XPS survey spectra. As it can be seen in Table 2, the synthesized materials contain C and O with a small amount of Si, probably due to the dust contamination during storage. The O/C ratio registered for the nanocomposites revealed a decreased value due to the change in elemental composition induced by addition of SW nanostructures; however, the SW-COOH containing materials have a higher value due to the cumulative effect of the functional groups from the reinforcing agent.

**Table 2:** Elemental composition of the ECO matrix and ECO-based nanocomposite materials

Sample	C % at	O % at	Si % at	O/C
ECO	71.2	23.7	5.1	0.332
ECO_SW1%	76.7	20.8	2.5	0.271
ECO_SW-COOH1%	67.4	26.1	6.5	0.387
ECO_SW-am1%	75.8	19.5	4.7	0.257

**Fig. 1:**
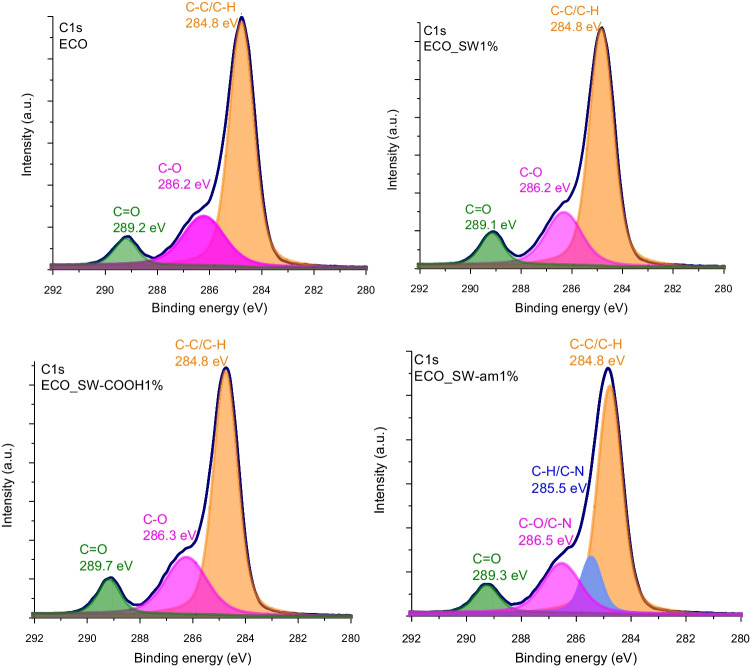
High-resolution C 1 s spectra from XPS

High-resolution XPS spectra were used to emphasize the C1s species from the bio-epoxy network as it can be observed from Fig. 1. The neat ECO network is composed by three C1s secondary peaks located at 284.8 eV, 286.2 eV, and 289.2 eV assigned to the carbonaceous species coming from C–C, C-O, and C = O bonds, respectively, present in the modified triglyceride structure as well as the citric acid curing agent. The additional ester linkages formed between the carboxyl functionalities present on the aromatic surface of carbon nanotubes and the epoxy matrix during the crosslinking process are evidenced by the increased area for C = O secondary peak in case of the ECO_SW-COOH1%. When looking at the nanocomposites reinforced with amide functionalized nanotubes, a new signal emerged from the C-N bonds.

Differential scanning calorimetry (DSC) was used to monitor the curing process through the curing reaction enthalpy evaluation during nonisothermal analysis with a heating rate of 5 K/min. The characteristics of the crosslinking mechanism with citric acid (AC) and the activation energy were calculated both by the Kissinger and Ozawa method for a comparison, leading to values close to 53 kJ/mol for the activation energy. According to Lascano et al. (2019) and Tudorachi and Mustata (2020), this value is similar to a conventional DGEBA type resin, denoting that the proposed polymeric matrix is a suitable candidate for coating applications.

Analysing the results presented in Table 3, one can observe that the addition of nano-reinforcing agents has a significant influence over the curing parameters. Looking at the curing enthalpy for the systems with neat SW, higher values in comparison with the ECO matrix were observed. A possible explanation may be an activation mechanism of COOH groups of AC exerted by the presence of the delocalized electrons from the SW aromatic surface. The studies undertaken by Zhiqiang Chen et al. (Chen et al. 2013) showed similar findings related to MWNT and conventional epoxy. Moreover, the exposed COOH and amide functional groups from the dispersed SW structures could disturb electron delocalization, leading to loss of this endowment. The proposed activation mechanism is also sustained by a lower Ea value calculated for the network formation through curing reaction.

**Table 3:** The DSC data calculated for ECO and the nanocomposites networks

Sample	(%)	ΔH (J/g)^*^	T_max_ (°C)^*^	Ea (kJ/mol) Kissinger	Ea (kJ/mol) Ozawa
ECO-AC		129.6	125.8	53.00	56.92
ECO_SW	0.25	135.0	129.6	39.12	43.76
	0.5	139.2	132.4	56.25	60.11
	1	151.8	120.1	46.64	50.82
ECO_SW-am	0.25	130.6	124.9	63.79	67.13
	0.5	112.7	126.1	53.66	57.56
	1	140.6	138.0	53.39	57.52
ECO_SW-COOH	0.25	102	125.9	57.18	60.89
	0.5	135.4	119.8	57.22	60.82
	1	92.32	128.5	59.39	63.03

^*Data resulted from the experiments recorded at 5 K/min heating rate^

The presence of NH and COOH functional groups on the surface of SW structures could provide a higher compatibility and a better dispersion between the bio-based epoxy matrix and the reinforcing agents. The average Ea values calculated for the ECO systems reinforced with amide functionalized SW are higher than the one for the neat matrix. These data suggest that instead of participating in the epoxy cleavage reaction, the amide groups could initiate a concurrent blocking interaction of the activated carboxyl groups from the curing agent. In the case of the SW-COOH addition, the activation energy is still slightly higher in comparison with the neat ECO system, and the enthalpy values are considerably lower than ECO matrix, suggesting that a percolation threshold for the nanotube content was reached between 0.25 and 0.5%. After this point, the cure reaction is slowed down by delayed diffusion processes of the curing agent. In a study conducted for conventional epoxy systems, Rezazadeh observed a steric hindrance exerted by the modified nanotubes during the cure process, leading to no significant improvement over the heat of reaction (Rezazadeh et al. 2017). In the same time, lower values given by some of the ECO-based formulations reinforced with functionalized SW are in accordance with this hypothesis.

**Fig. 2:**
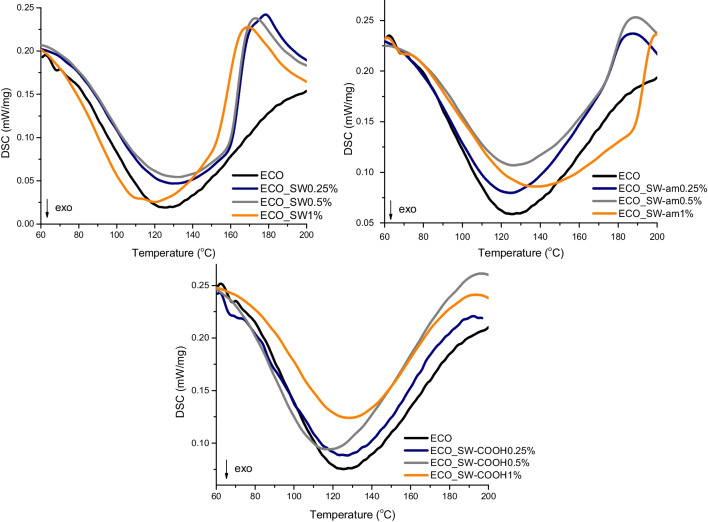
DSC curves registered for the ECO systems reinforced with SW (**a**), SW-am (**b**), and SW-COOH (**c**) recorded at 5 K/min heating rate

The curing profiles from the DSC curves (Fig. 2) are valuable to further correlate the Ea values and the hypothesis concerning the higher enthalpy issued above. Thus, the ECO-based systems with SW display a wide peak with a shoulder. The latter is assigned to the secondary thermal events generated by the physical interactions exerted between the aromatic structures of the nanotubes and the carboxyl groups of the curing agent. This is not visible within the curves registered for the SW-COOH nanocomposites, which are symmetrical and narrower, probably due to the homogeneous type of functional groups within the formulation.

Correlating the information revealed by the DMA data comprised in Table 4, it can be noticed that the glass transition temperature has values close to RT (~ 25 °C), which correspond to elastomer type materials with high damping properties. The addition of neat SW not only lowers the Tg values, but also affects the crosslinking density through a hindering effect, thus minimizing the interactions between COOH and epoxy towards curing. However, the presence of physical interactions noticed in the DSC results among the aromatic structure of the nanotubes and the COO^−^ functionalities are not excluded by the DMA data. In case of amide functionalized SW nanocomposite systems, an increase in both Tg and CD values is observed, probably due to the formation of a secondary network within the ECO matrix as a consequence of a better compatibility between the components.

**Table 4:** DMA experimental results for ECO matrix and its nanocomposites

Sample	(%)	Tg (°C)	CD (mol/cm^3^)	E′ at 25 °C (MPa)
ECO-AC		25.6	2175	133.7
ECO_SW	0.25	18.7	1782	34.2
	0.5	22.2	1565	75.6
	1	20.2	1642	34.5
ECO_SW-am	0.25	29.4	2043	32.1
	0.5	24.1	2351	57.2
	1	31.4	2124	173.5
ECO_SW-COOH	0.25	17.8	1492	247.4
	0.5	20.3	2345	100.1
	1	28.1	3424	288.2

The influence of the content of carboxyl functional groups from the SW surface is only noticeable when 1% nanocomposites are analysed, as it was observed also by the increased C/O ratio and the higher area assigned to C = O species from XPS results. The specific mechanism which underlies the enhancement of mechanical performance and crosslinking density in the SW-COOH reinforced systems may be caused by the reactivity of the functional groups, which can be engaged in the bio-epoxy network formation. Considering that also the curing process is relying on carboxylic acid attack, a homogeneous distribution of SW-COOH within the bio-based is expected as the result of a good compatibility with the bio-epoxy matrix. Thus, the highest values for CD and E′ denote a more rigid material, with a slightly higher Tg compared to the matrix (Chee et al. 2017; Raditoiu et al. 2014).

**Fig. 3:**
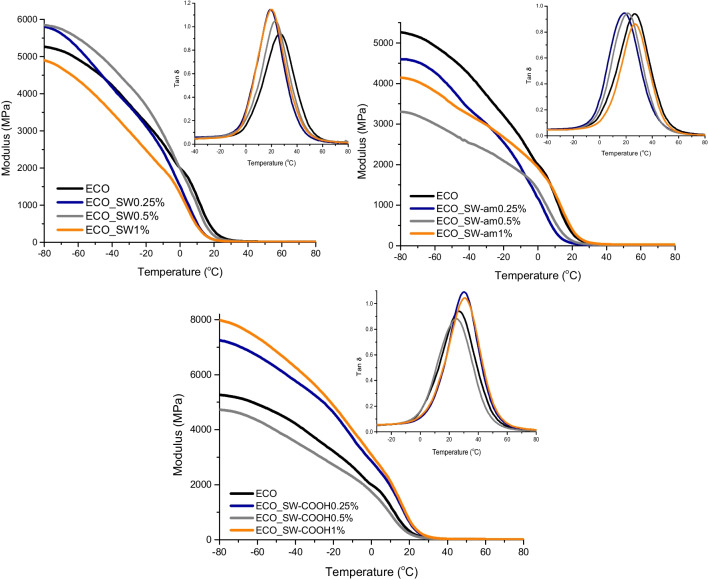
DMA profiles of the ECO-based nanocomposites

A homogeneous distribution of the nano-reinforcing agent within the ECO matrix is also sustained by the allure of the tan δ peaks (Fig. 3), which display a similar profile to the ECO sample, suggesting that the presence of the carbonaceous structures is not disturbing the integrity of the network. Apart from that, the transition between the rigid glassy states highlighted by the storage modulus plateau varies as a function of the surface chemistry of the SWs. Thus, when neat SW is used, a narrow transition is observed, while in the case of amide and carboxyl-functionalized SWs, it broadens. These findings sustain the results from DSC analysis, according to which the nano-reinforcing agents are reactive towards the crosslinking process, leading to different conducts for the macromolecular segments between the crosslinking points.

**Fig. 4:**
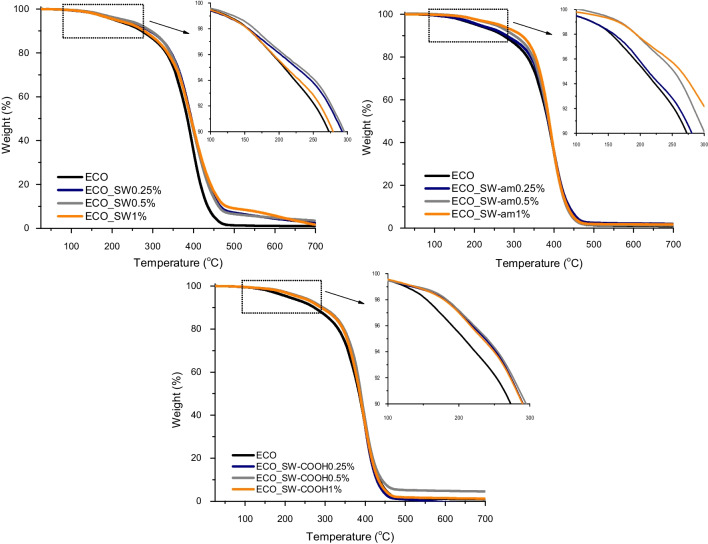
TGA profiles of ECO_SW-based nanocomposites

**Fig. 5:**
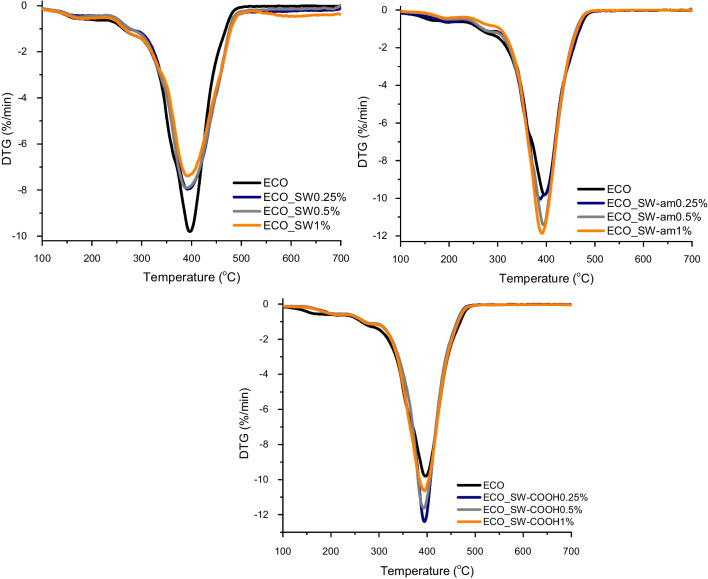
DTG profiles of ECO_SW-based nanocomposites

The thermal behaviour of the nanocomposites with ECO matrix reinforced with different types of neat and functionalized SWs was evaluated by TGA, and the corresponding thermograms are presented in Fig. 4 and 5. As it is already known, carbon nanotubes exhibit superior thermal properties (Ruoff and Lorents 1995). This feature was successfully translated to the newly synthesized nanocomposites, where considerable higher thermal stability was revealed according to the data presented in Table 5. In case of the nanocomposites reinforced with neat SWs, an increase with more than 40 °C in the values of T_d3%_ is noticed, while the addition of amide and carboxyl-functionalized SWs to the ECO network improves thermal stability with more than 80 °C and 60 °C, respectively, which is clear evidence that the nanotubes are generating a set of bio-based materials with enhanced thermal characteristics, as it can be noticed for the T_d5%_ and T_d10%_ values. Nonetheless, when the maximum degradation rate temperature is monitored, it can be seen that the values of all systems are similar, highlighting once again the findings from DMA results according to which the epoxy networks which are formed have homogeneous behaviour. A hypothesis regarding this conduct could be assigned to the formation of a secondary network by the SW structures with much higher thermal and mechanical properties. These features of the nano-reinforcing agents are translated efficiently to the ECO matrix, leading to a synergic effect by generating a protective thermal barrier exerted on the coated substrate.

**Table 5:** TGA data obtained for the ECO matrix and its nanocomposites with SWs

Sample	(%)	T_d3%_ (°C)	T_d5%_ (°C)	T_d10%_ (°C)	T_max_ (°C) from DTG	Residual mass (%)
ECO-AC		137.4	207.3	273.1	396.1	0.95
ECO_SW	0.25	179.0	224.6	292.5	394.0	2.43
	0.5	184.8	231.2	295.4	389.5	3.53
	1	173.7	210.1	279.2	393.1	1.62
ECO_SW-am	0.25	180.8	212.7	280.8	386.8	2.03
	0.5	213.4	252.7	301.0	394.0	1.30
	1	218.0	264.0	319.1	390.8	1.70
ECO_SW-COOH	0.25	203.5	237.9	291.8	394.7	1.14
	0.5	203.5	239.8	294.7	393.4	4.55
	1	200.0	233.5	289.9	392.1	1.19

**Fig. 6:**
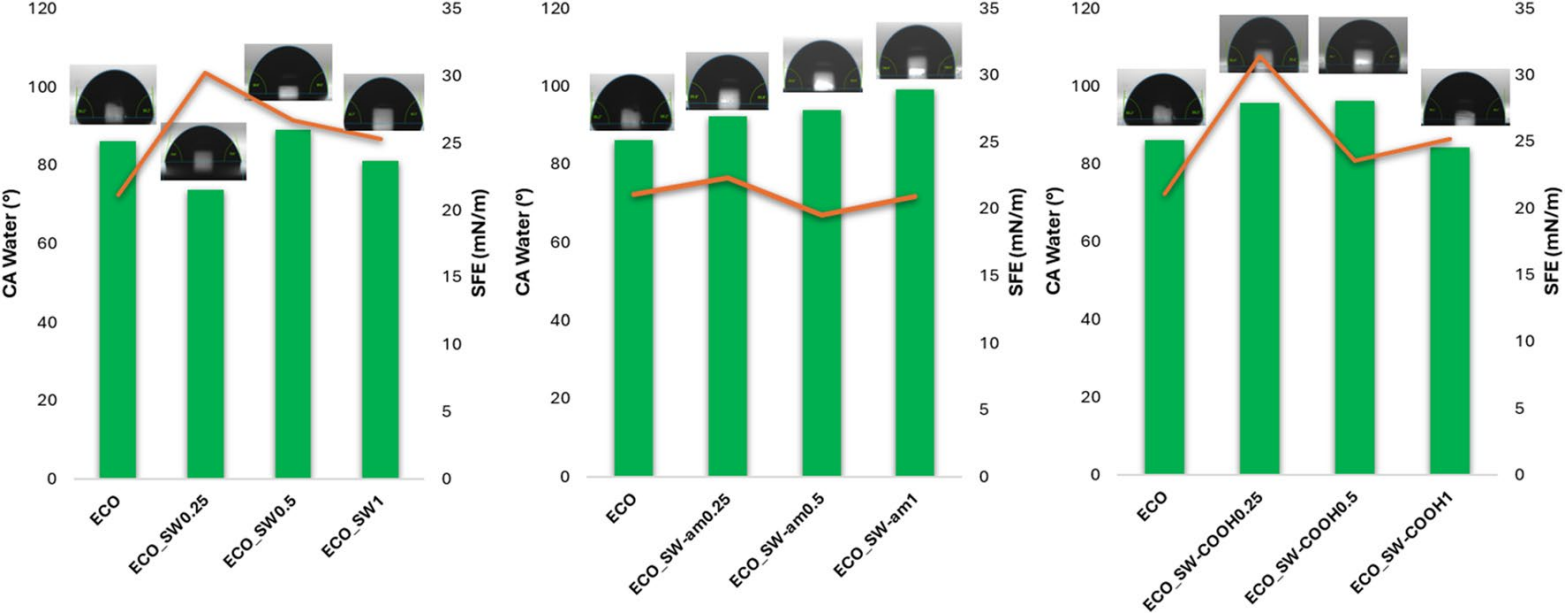
Surface characteristics for ECO-SW-based nanocomposites

The wettability of the ECO-based materials surface was assessed by contact angle (CA) measurements, and the corresponding results are represented in Fig. 6. These preliminary results are in accordance with the envisaged coatings application. According to CA results, an ideal formulation for a protective coating is represented by the SW-COOH nanocomposites which not only possess a hydrophobic character brought by the CNT aromatic structure, but also has superior values for SFE which suggest a good compatibility with wide range of substrates. The decreased value for water CA in case of ECO_SW-COOH1% indicates that the percolation threshold for SW-COOH content was reached between 0.5 and 1%; as a consequence, the hydrophilic-hydrophobic balance within the material was altered. On the other hand, the composites containing 0.25 and 0.5% SW-COOH showed high water CA values, sustaining thus the formation of a more compact network by involving more COOH reactive sites in the cure process, as demonstrated also by the higher CD values from DMA.

**Fig. 7:**
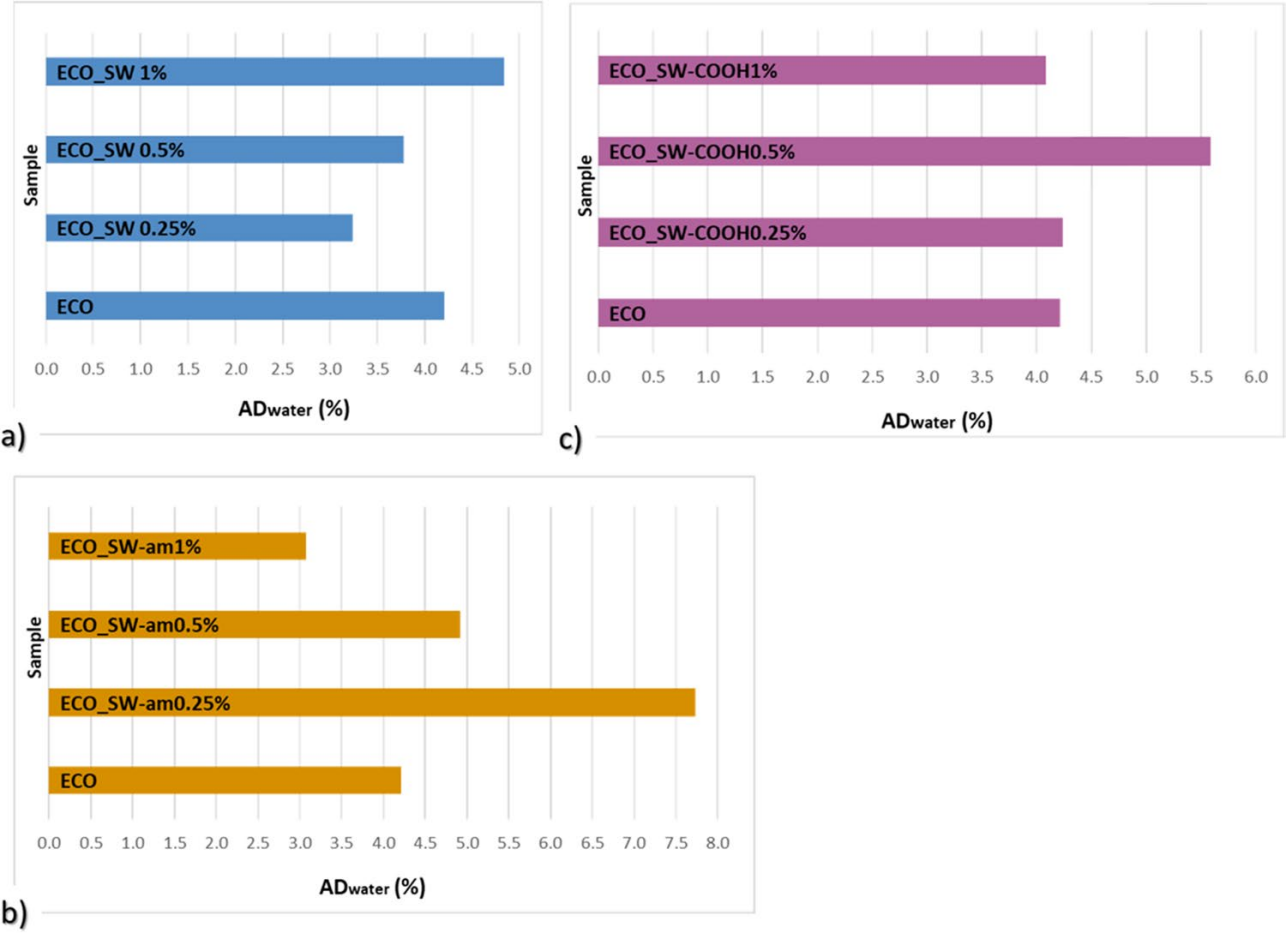
Water absorption degree of ECO-based composites bearing different SW species: **a** neat SW, **b** SW-am, **c** SW-COOH

**Fig. 8:**
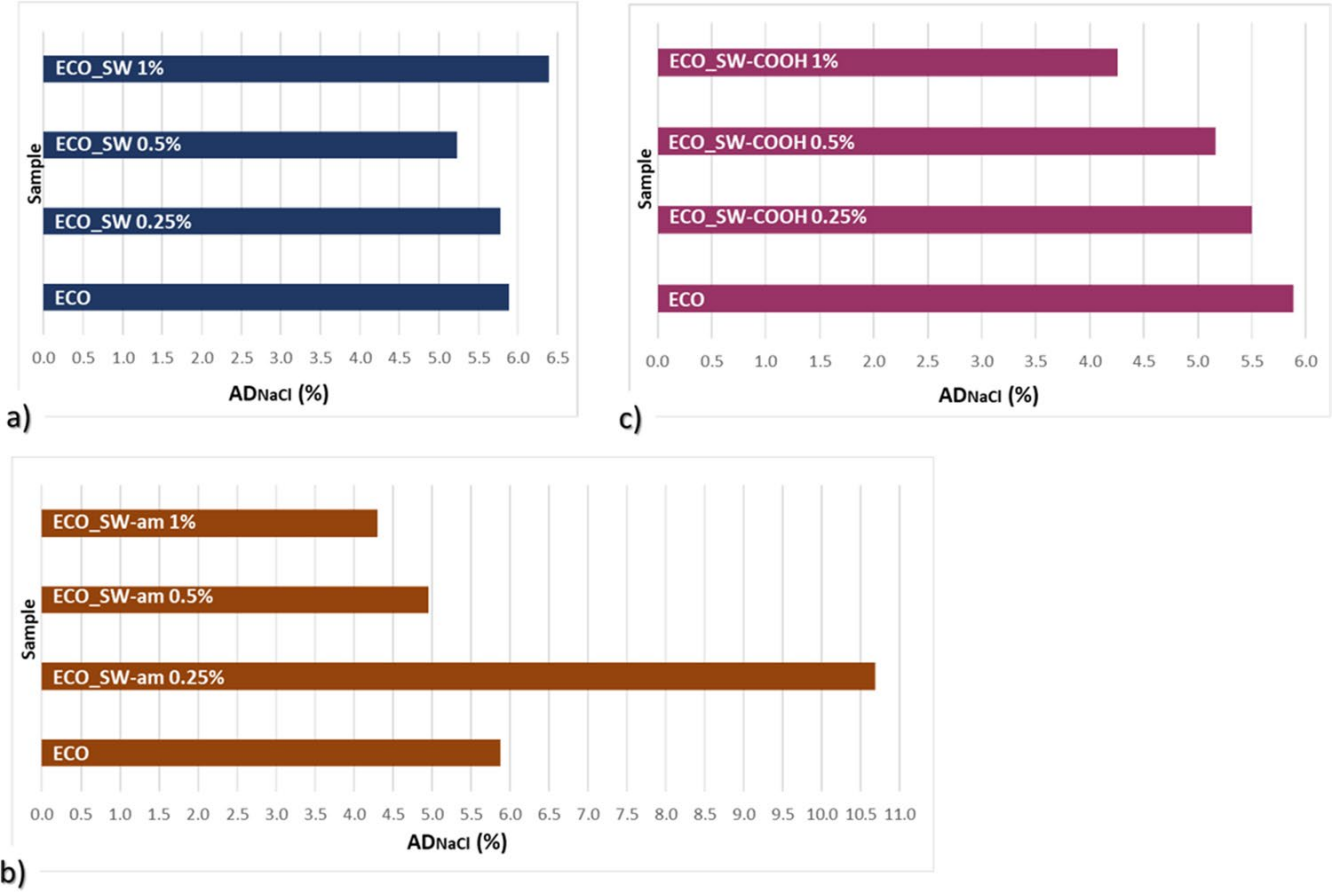
Seawater absorption degree of different ECO-SW composites, containing **a** neat SW, **b** SW-am, **c** SW-COOH

In the context of the impact upon the environment and their lifetime, all the synthesized ECO-based polymer composites were evaluated in terms of their affinity for aqueous media: water and seawater (as simulated seawater, with a salt concentration of 3.5% (Nasreen and Ijmtst 2022). The results of water/NaCl solution absorption degree (AD_water_/AD_NaCl_) are graphically represented in Fig. 7 and 8, where each type of composite system (ECO loaded with SW or SW-am or SW-COOH) is compared with the polymer matrix (ECO). It is well known that the aqueous media affects the macro- and micro-structure of the composite matrices. This is a big concern of the manufacturers of materials with technical applications in various industries (Barreira-Pinto et al. 2023). While different reinforcing agents are loaded to improve the strength of the materials, the continuous phase (polymer matrix) plays the protective role for the covered surface.

The starting point of water and seawater absorption considers the effect of the reinforcing agents on the improvement of the materials strength, while maintaining the anticorrosion protective role of the ECO polymer network by increasing hydrophobicity.

When looking to the obtained nanocomposites, the addition of neat nanotubes in small quantities lead to lower AD_water_ values which is a good point in the discussed context, but at higher amounts, the addition of this filler induces a higher water retention capacity, which disadvantages a protective coating, potentially leading to its degradation in a shorter time.

The coating materials synthesized from ECO reinforced with amide functionalized SW revealed inconsistent behaviour regarding water absorption, with fluctuations of the different reinforcement proportions that are difficult to argue. Immersion in water indicates the best conduct for the specimens loaded with SW-COOH, with a slightly lower water affinity in comparison with the ECO matrix (Fig. 7c). This can be evidence of carboxyl species from the SW surface involvement in the crosslinking reaction under temperature, acting as supplementary crosslinking agent for the bio-based epoxy resin. This result is well correlated with the higher CD value calculated for this ECO_SW-COOH system (3424 mol/cm^3^), which is also highlighted by the DSC results. From the point of view of the material behaviour, the ECO material containing 1% SW-COOH has a packed structure which is translated as reduced free volume, and thus driven by the hydrophobicity of the ECO matrix, the penetration of water molecules towards protected substrate is difficult.

Also, in the case of seawater absorption, the specimens containing different proportions of SW-COOH filler indicate the best and consistent behaviour regarding resistance in the aquatic environment. In this case, the higher the functionalized SW ratio, the material resistance is better. Comparing the affinity for saline solution of neat ECO with ECO_SW-COOH specimens, it was noticed an improvement of up to 28% in terms of decreasing AD values when 1% SW-COOH was added. In this case, a secondary curing process which is conducted by the carboxyl groups from the SW is sustained also by other studies (Sales-Contini et al. 2023).

**Fig. 9:**
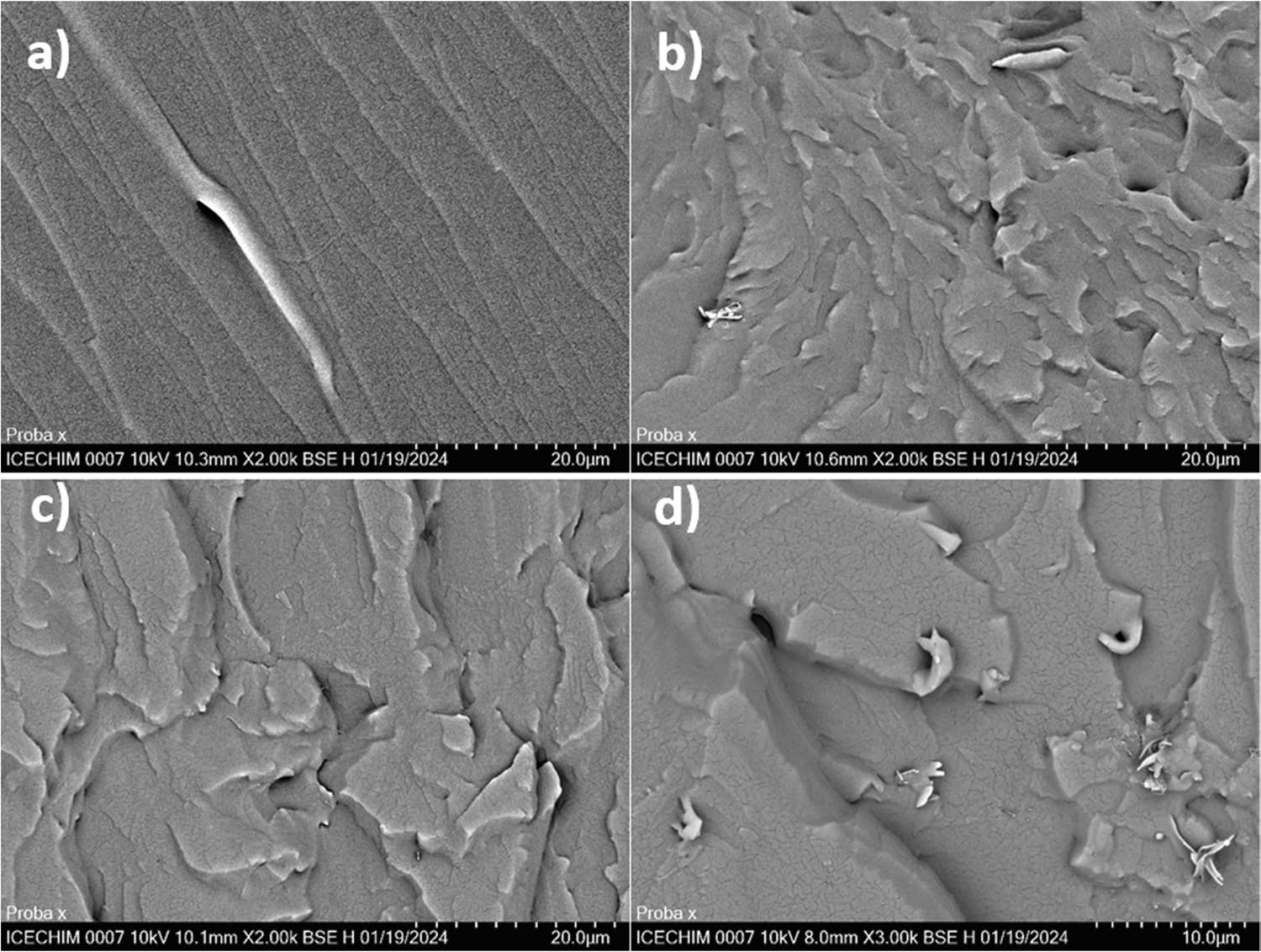
SEM micrographs of fracture morphology for ECO matrix (**a**), the nanocomposites with SW (**b**), SW-COOH (**c**), and SW-am (**d**)

To assess the dispersion of the nano-reinforcing agent within the polymeric epoxy matrix, SEM analysis was performed, and the resulting images are displayed in Fig. 9. Neat ECO matrix morphology revealed a smooth fracture surface, according to a homogeneous network without segregation of the phases during the curing process. However, as it was expected, due to the flexible nature of the triglyceride chains, a ductile feature can be noticed as suggested by the linearity of the cracks along with the elongation of the highlighted area. The surface roughness of the nanocomposites with 1% reinforcing agents is strongly influenced by the functionality of the SW structures. Thus, the neat SW surface promotes the agglomeration of these structures within the ECO matrix, leading to prominent edges of the nanocomposite fracture as seen in Fig. 9b.

An effective dispersion of the SW-COOH within the ECO system was observed in Fig. 9c as displayed by the homogeneity of the composition as a consequence of the higher compatibility between the functional groups from the nano-reinforcing agent surface and the matrix. In contrast, when analysing Fig. 9d, the SW-am nanostructures led to notable agglomerates, probably due to different polarities between the components of the nanocomposites.

## Conclusions

The present study highlights the impact of different types of SWs over the structural, thermal, and surface properties of epoxidized corn oil nanocomposites, offering a glimpse into their potential applications as protective coatings against corrosion.

According to DSC results, the addition of nano-reinforcing agents influences the curing parameters of the newly developed networks. The additional amide and COOH functionalities present on the surface of the CNT interact with the bio-based epoxy matrix leading to stronger networks with better properties. The lower enthalpy values for the 1% SW-COOH nanocomposites indicate a critical concentration of available functional groups reached between 0.5 and 1% as demonstrated also by the water CA results.

The lower values for Tg indicate elastomer-type materials with high damping properties with a homogeneous distribution of nano-reinforcing agents within the ECO matrix as demonstrated by the Tan δ profiles. Neat SW lowers Tg values and affects crosslinking density, while SW-am and SW-COOH show increased Tg and crosslinking density reiterating thus the participation of the functionalities of CNT in the formation of the epoxy network.

Epoxidized corn oil SW’s nanocomposites showed remarkable improvement in thermal properties. ECO_SW-am-1 showed an increase in T_d3_% with ~ 80 °C as compared with ECO matrix, while ECO_SW-COOH samples showed an increase in thermal stability with ~ 60 °C. In the case of SW-COOH nanocomposites, similar thermal properties were observed independent of the reinforcing agent ratio.

Evaluation of water and seawater absorption indicates that the incorporation of neat SWs in small quantities leads to considerably lower water absorption. SW-COOH nanocomposites show consistent and improved resistance to water and seawater, suggesting a protective role in the ECO polymer network.

## Data Availability

All data comprised within the study are available upon request.
